# Management of Gigantomastia: Outcomes of Superomedial Pedicle with Vertical Scar or Wise Pattern Skin Excision 

**Published:** 2017-05

**Authors:** Mehmet Can Sak, Selcuk Akın, Burak Ersen, Orhan Tunalı, Aksu Ismail

**Affiliations:** 1 Bıngol State Hospıtal, Plastıc Reconstructıve and Aesthetıc Surgery Department, Bingol, Turkey;; 2 Uludag University, Faculty Of Medıcıne, Plastıc Reconstructıve and Aesthetıc Surgery Department, Uldag, Turkey;; 3 Kassatıon State Hospıtal, Plastıc Reconstructıve and Aesthetıc Surgery Department, Kassation, Turkey

**Keywords:** Gigantomastia, Breast, Skin, Excision, Pedicle, Scar

## Abstract

**BACKGROUND:**

Gigantomastia is a rare condition characterized by excessive breast growth and can be physically and psychosocially disabling for the patient. Regarding management of gigantomastia, this study evaluates the outcomes of superomedial pedicle with vertical scar or wise pattern skin excision.

**METHODS:**

A total of 425 patients who underwent reduction mammoplasty in our institution were reviewed. Forty eight reduction mammoplasty patients with resection weights greater than 1 kg per breast and treated with superomedial dermoglandular pedicle technique combined with vertical or wise-pattern skin excision were included.

**RESULTS:**

The patients were between 19 and 66 years old, with an average of 41 years. Total weight of resection was between 1000 and 2600 g, with an average of 1384 grams for right breast and between 1000 and 3000g, with an average of 1434 grams for left breast. The secondary revisions and wound healing complications were extremely high in vertical scar group compared to wise pattern group (87,5% and 12,5%, respectively).

**CONCLUSION:**

The authors concluded that superomedial dermoglandular pedicle in the addition of a wise pattern is an appropriate, safe and reliable method when dealing with significantly larger breasts (>1000g).

## INTRODUCTION

Gigantomastia is a rare condition characterized by excessive breast growth and can be physically and psychosocially disabling for the patient. To date, there is no universal classification or accepted definition for gigantomastia. Many authors cite gigantomastia as breast enlargement that requires reduction of over 1500 g per breast. However, there is discordance in the literature with the weight of reduction ranging from 800 to 2000 g.^1^ Reduction mammoplasty in patients with gigantomastia can prove a challenge for the plastic surgeon. Various techniques can be used to reduce mild to moderately large breasts. However, the ideal reduction method for severe gigantomastia cases (1000 g per breast reduction) remains controversial. Therefore, most of the authors still prefer the “free nipple” technique.^2^ The disadvantage of the technique is flat, non-projecting, and insensate nipples. In addition to this, partial take of the graft leads to irregularly pigmented areas, which are particularly obvious in the darker skinned patients. Although this is not a new technique, the acceptable reduction mass remains uncertain.^3^

In breast reduction, techniques that provide safe and predictable results with nipple preservation are preferred.^4^ Dermoglandular pedicle techniques are now used routinely; however, the ideal technique for preserving the nipple-areola complex during breast reduction in gigantomastia patients is still arguable.^2^ Various procedures have been described for reduction mammoplasty with specific skin incisions, patterns of breast parenchymal resection, and retained blood supply to the remaining breast tissue and areolar complex; however, not all of these techniques can be applied successfully in the setting of gigantomastia.^5^ This retrospective study aims to analyze the outcomes of reduction mammoplasty for gigantomastia using the superomedial dermoglandular technique combined with verticalorwise-pattern skin excision.

## MATERIALS AND METHODS

Data were collected over a 5 year period from 2008 to 2013. A retrospective review was performed of 425 patients who underwent reduction mammoplasty in our institution. From these patients, resection weights smaller than 1 kg per breast and treated with free-nipple graft technique or other dermoglandular pedicle techniques weree xcluded. Forty eight reduction mammoplasty patients with resection weights greater than 1 kg per breast and treated with superomedial dermoglandular pedicle technique combined with verticalorwise-pattern skin excision were included in the study. Patients were randomly selected for each technique. Data on these patients was collected retrospectively, and patient demographics, resection weights, complications and reoperation reasons in postoperative one year were recorded. All operations were performed by same surgeon (MCS).

## RESULTS

The patients included in this study were between 19 and 66 years old, with an average of 41 years. Total weight of resection (grams per side) was between 1000 and 2600 g, with an average of 1384 grams for right breast and was between 1000 g and 3000 g, with an average of 1434 grams for left breast. Operations were performed by different surgeons under general anesthesia. Twenty four patients were operated by using superomedial dermoglandular pedicle in combination with the vertical scar technique (Table 1 and 2). The written informed consent is taken for each patient. 

## DISCUSSION

Reduction mammoplasty is a reconstructive procedure performed for the alleviation of pain and discomfort associated with excessive and pendulous breast tissue of any origin. Throughout the historical evolution of techniques, many surgeons pioneered different procedures as the understanding of breast anatomy flourished and patient’s expectations for aesthetically pleasing results and minimal scaring increased. Breast reduction are performed in women with excessive breast tissue who present with any of these associated symptom: head, neck, shoulder and back pain; brassiere strap groove caused by a tight-fitting brassiere; limitation of activities of daily living; intertrigimous dermatitis; sleep disturbances; and/or respiratory problems. Also, significant psychosocial sequel associated with large breasts cannot be overlooked.^6^

The superomedially basedpedicle was first described by Orlando and Guthrie in 1975 for reduction mammoplasty.^7^ The choice of skin and glandular resection patterns in combination with this pedicle can vary according to the amount and quality of the excess skin andgland. To reduce aesthetic complications, adaptations of the Hall-Findlay vertical reduction with medial or superomedial pedicles have recently gained acceptance.^8^ The superomedial pedicle with vertical scar reduction allows for a shorter scar with decreased scar hypertrophy, as well as the benefits of retained upper pole fullness and more extensive lateral parenchymal reduction, producing a desirable surgical result with greater projection.^9^

While the superomedial pedicle with vertical scar reduction technique has proven effective for small and medium volume reductions, some surgeons have expressed hesitancy in applying the superomedial pedicle with vertical scar reduction techniques for large-volume reduction mammoplasties, citing increased complications rates with higher resection volumes.^3^^,^^10^^,^^11^ The author performed superomedial pedicle with vertical scar reduction technique in 24 patients as mentioned at Table 1. The highest complication and reoperation rates were noted in these patients due to excessive pedicle length as well as torsion, twisting, and compression of the pediclefor ensuring vertical scar. In this report of 24 patients following superomedial pedicle with vertical scar reduction mammoplasty, nipple-areola viability was demonstrated in 21 patients. 

**Table 1:** Patiens treated with superomedial dermoglandular pedicle and wise pattern excision

**Patients**	**Age**	**R**	**L**	**Complications and reoperation reasons**
1	26	2600g	3000g	None
2	42	1730g	1745g	None
3	50	1400g	1440g	None
4	47	1200g	1100g	None
5	48	1240g	1150g	Needs reoperation due to hypertrophic scar formation around areola.
6	48	1300g	1100g	None
7	53	1330g	1100g	None
8	46	1580g	1200g	None
9	46	1700g	1500g	None
10	41	1135g	1135g	None
11	55	1340g	1320g	Needs reoperation due to fat necrosis in left breast.
12	42	1500g	1450g	None
13	30	1200g	1500g	None
14	51	1330g	1220g	None
15	30	1500g	1900g	Needs reoperation due to left nipple areola complex necrosis.
16	29	1650g	1550g	None
17	30	1150g	1100g	None
18	46	1165g	1100g	None
19	43	1250g	1100g	None
20	38	1700g	2000g	None
21	32	1130g	1100g	None
22	37	1325g	1225g	None
23	35	1300g	1300g	None
24	38	2000g	2000g	None

R: Weight of excision from right breast; L: Weight of excision from left breast

**Fig. 1:**
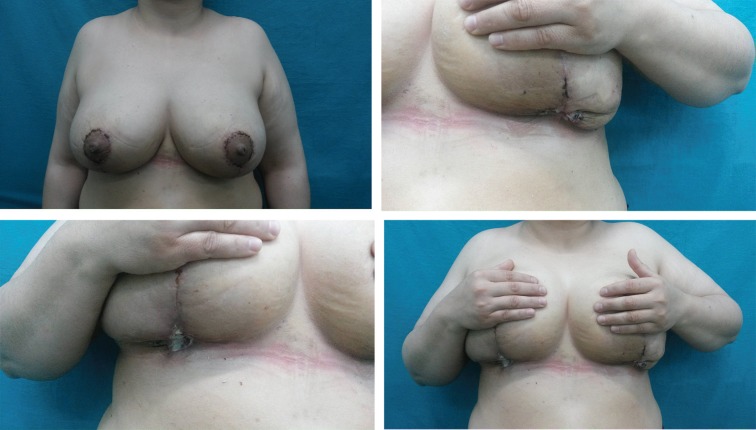
The major complication of wound dehiscence at the purse string closure sites that healed with secondary intention.

The other reasons of reoperations in this group were double bubble deformity due to inadequate resection seen in 3 patients; wound dehiscence at the purse string closure sites of infra mammarian folds seen in 45 patients. The major complication was wound dehiscence at the purse string closure sites that would be healed with secondary intention (Figure 1a-d). The authors found that resections as large as 2500 g were well tolerated with nipple viability by superomedial vertical scar reduction but due to high complications and reoperation rates they have left this technique after wards and pioneered superomedial pedicle with wise pattern skin excision. The author performed superomedial pedicle with wise pattern reduction technique inanother 24 patients as mentioned at Table-2. 

**Table 2:** Patiens treated with superomedial dermoglandular pedicle and vertical scar excision pattern

**Patients**	**Age**	**R**	**L**	**Complications and reoperation reasons**
1	49	1810g	1750g	Needs reoperation due to “double-bubble” deformity observed at inframammarian folds of each breast.
2	33	1200g	1100g	Needs reoperation due to wound dehiscence at purse-string closure sites of each breast
3	47	1200g	1300g	Wound dehiscence at purse-string closure sites. No need for reoperation. Heals with secondary intention.
4	35	1300g	1420g	Wound dehiscence at purse-string closure sites. No need for reoperation. Heals with secondary intention.
5	26	1100g	1100g	Needs reoperation due to left nipple areola complex necrosis.
6	27	2500g	2250g	Needs reoperation due to wound dehiscence at purse-string closure sites of each breast
7	45	1150g	1200g	Needs reoperation due to “double-bubble” deformity observed at inframammarian folds of each breast.
8	39	1410g	1335g	Wound dehiscence at purse-string closure sites. No need for reoperation. Heals with secondary intention
9	59	1100g	1100g	Needs reoperation due to wound dehiscence at purse-string closure sites of each breast
10	39	1200g	1500g	Needs reoperation due to wound dehiscence at purse-string closure sites of each breast
11	42	1600g	1725g	Wound dehiscence at purse-string closure sites. No need for reoperation. Heals with secondary intention
12	51	1000g	1000g	Double bubble deformity. Needs additional resection due to asymmetry and large breasts. 320gr additional tissue from right breast and 480gr additional tissue from left breast were removed using same procedure nine months later.
13	47	1150g	1300g	None.
14	38	1400g	1450g	Needs reoperation due to hematoma formation and wound dehiscence at purse-string closure sites of each breast
15	34	1100g	1600g	Wound dehiscence at purse-string closure sites. No need for reoperation. Heals with secondary intention
16	36	1300g	1200g	Wound dehiscence at purse-string closure sites. No need for reoperation. Heals with secondary intention
17	45	2100g	2500g	Needs reoperation due to right nipple areola complex necrosis and wound dehiscence at purse-string closure sites of each breast
18	41	1200g	1200g	None.
19	66	1135g	1300g	None.
20	19	1150g	1150g	Wound dehiscence at purse-string closure sites. No need for reoperation. Heals with secondary intention.
21	26	1705g	1605g	Wound dehiscence at purse-string closure sites. No need for reoperation. Heals with secondary intention.
22	33	1700g	1700g	Needs re-operation due to bilateral nipple areola complex necrosis
23	40	1200g	1600g	Wound dehiscence at purse-string closure sites. No need for reoperation. Heals with secondary intention.
24	30	1230g	1100g	Wound dehiscence at purse-string closure sites. No need for reoperation. Heals with secondary intention.

R: Weight of excision from right breast; L: Weight of excision from left breast.

The authors found that resections as large as 3000 g were well tolerated with nipple viability by superomedial pedicle with wise pattern scar reduction. The overall complications and secondary revisions in these patients were dramatically decreased when compared to superomedial pedicle vertical scar reduction technique. These operation reasons were hypertrophic scar formation in onepatient; fat necrosis in one patient and nipple areola complex necrosis in onepatient. Wound healing issues along the T-junction, have not been observed in anycase. The authors noted less complications postoperatively due to freemovement of pedicle in these patients. Recent cadaveric studies have shown that superomedial based pedicles capture the main venous outflow of the nipple areola complex, which drains directly into the internal mammary veins at the level of the second and third inter-costal perforators.^11^

In our study, there were four episodes of nipple necrosis in 48 patients with a rate of 8.3% which is higher compared to similar studies. Spear *et al.*^12^ havereported nipple necrosis rates of 3.6% when performing a Le jour type vertical reduction with a superomedial based pedicle. But Spear *et al.* emphasized in their survey that their technique is applicable to younger, nonobese patientswith small to moderate breast reductions (size under 1000 g), with adequate skin elasticity and minimal to moderate associated ptosis. Traditionally, the vertical closure has resulted in significant gathering of the breast skin and subsequent pleating that often led to secondary revisions, with rates ranging between 7% and 20%.^13^^,^^14^

In this study, the authors found that the secondary revisions and wound healing complications were extremely high in vertical scar group compared to wise pattern group, 87,5% and 12,5% respectively. The authors concluded that superomedial dermoglandular pedicle in the addition of a wise pattern is an appropriate, safe and reliable method when dealing with significantly larger breasts (>1000 g).

## CONFLICT OF INTEREST

The authors declare no conflict of interest.
